# Neuropsychiatric Ramifications of Severe COVID-19 and Other Severe Acute Respiratory Infections

**DOI:** 10.1001/jamapsychiatry.2022.1067

**Published:** 2022-05-11

**Authors:** Ashley Kieran Clift, Tom Alan Ranger, Martina Patone, Carol A. C. Coupland, Robert Hatch, Karen Thomas, Julia Hippisley-Cox, Peter Watkinson

**Affiliations:** 1 Nuffield Department of Primary Care Health Sciences, University of Oxford, Oxford, United Kingdom; 2 Cancer Research UK Oxford Centre, University of Oxford, Oxford, United Kingdom; 3 Division of Primary Care, School of Medicine, University of Nottingham, United Kingdom; 4 Nuffield Department of Clinical Neurosciences, University of Oxford, Oxford, United Kingdom; 5 Intensive Care National Audit and Research Centre, London, United Kingdom; 6 Oxford Biomedical Research Centre, Oxford University Hospitals NHS Trust, Oxford, United Kingdom

## Abstract

**Importance:**

Individuals surviving severe COVID-19 may be at increased risk of neuropsychiatric sequelae. Robust assessment of these risks may help improve clinical understanding of the post–COVID syndrome, aid clinical care during the ongoing pandemic, and inform postpandemic planning.

**Objective:**

To quantify the risks of new-onset neuropsychiatric conditions and new neuropsychiatric medication prescriptions after discharge from a COVID-19–related hospitalization, and to compare these with risks after discharge from hospitalization for other severe acute respiratory infections (SARI) during the COVID-19 pandemic.

**Design, Setting, and Participants:**

In this cohort study, adults (≥18 years of age) were identified from QResearch primary care and linked electronic health record databases, including national SARS-CoV-2 testing, hospital episode statistics, intensive care admissions data, and mortality registers in England, from January 24, 2020, to July 7, 2021.

**Exposures:**

COVID-19–related or SARI-related hospital admission (including intensive care admission).

**Main Outcomes and Measures:**

New-onset diagnoses of neuropsychiatric conditions (anxiety, dementia, psychosis, depression, bipolar disorder) or first prescription for relevant medications (antidepressants, hypnotics/anxiolytics, antipsychotics) during 12 months of follow-up from hospital discharge. Maximally adjusted hazard ratios (HR) with 95% CIs were estimated using flexible parametric survival models.

**Results:**

In this cohort study of data from 8.38 million adults (4.18 million women, 4.20 million men; mean [SD] age 49.18 [18.45] years); 16 679 (0.02%) survived a hospital admission for SARI, and 32 525 (0.03%) survived a hospital admission for COVID-19. Compared with the remaining population, survivors of SARI and COVID-19 hospitalization had higher risks of subsequent neuropsychiatric diagnoses. For example, the HR for anxiety in survivors of SARI was 1.86 (95% CI, 1.56-2.21) and for survivors of COVID-19 infection was 2.36 (95% CI, 2.03-2.74); the HR for dementia for survivors of SARI was 2.55 (95% CI, 2.17-3.00) and for survivors of COVID-19 infection was 2.63 (95% CI, 2.21-3.14). Similar findings were observed for all medications analyzed; for example, the HR for first prescriptions of antidepressants in survivors of SARI was 2.55 (95% CI, 2.24-2.90) and for survivors of COVID-19 infection was 3.24 (95% CI, 2.91-3.61). There were no significant differences observed when directly comparing the COVID-19 group with the SARI group apart from a lower risk of antipsychotic prescriptions in the former (HR, 0.80; 95% CI, 0.69-0.92).

**Conclusions and Relevance:**

In this cohort study, the neuropsychiatric sequelae of severe COVID-19 infection were found to be similar to those for other SARI. This finding may inform postdischarge support for people surviving SARI.

## Key Points

**Question:**

What are the risks of neuropsychiatric disorders in adults surviving COVID-19 hospitalization, and how do these compare with non-COVID severe respiratory infections?

**Findings:**

In this cohort study of data from more than 8 million adults in England, during the COVID-19 pandemic, risks of new anxiety disorder, dementia, psychotic disorder, and bipolar disorder diagnoses were significantly increased in adults surviving hospitalization for COVID-19 or other severe acute respiratory infections compared with the general population. Risks of neuropsychiatric illnesses or commencement of related medications were similar for COVID-19 and non-COVID severe respiratory infections.

**Meaning:**

The results of this study suggest that disease severity, rather than pathogen, is a relevant factor associated with neuropsychiatric ramifications after severe respiratory infections.

## Teaser

This cohort study of electronic health care data of more than 8 million individuals in England attempts to quantify the neuropsychiatric sequelae following discharge from COVID-19 hospitalization compared with patients surviving hospitalization due to non–COVID-19 severe acute respiratory infections.

## Introduction

The COVID-19 pandemic has caused over 5 million deaths worldwide and predicated profound social and economic disruption.^1^ There is continued interest in various conditions and the risk of severe outcomes due to SARS-CoV-2 infection and increasing interest in its long-term effects. Although the postacute COVID-19 syndrome is poorly understood, there are clinically relevant postinfection outcomes associated with several organ systems.^2,3^

Prepandemic data suggest that individuals with severe acute respiratory infections (SARI) have increased risks of subsequent neuropsychiatric illness and cognitive impairment compared with the general population.^4,5^ Furthermore, previous SARS virus outbreaks have been associated with notable burdens of neuropsychological sequelae.^6^ Recent evidence from the US suggests that the risk of such sequelae after COVID-19 of any severity may be significantly higher than that observed after influenza, or acute respiratory infections of any kind, during the time of the COVID-19 pandemic.^7^ However, such comparisons with influenza have limited follow-up, have not considered medication use, and may be problematic due to differential disease severity and atypical influenza dynamics.^8^ Other recent studies in England examining posthospitalization sequelae must be considered in terms of limited sample size and follow-up, and their focus on patient-reported, physiological, and biomarker assays without quantifying formal diagnoses and medication use.^9^ The association between COVID-19 and neuropsychological sequelae, therefore, requires further clarification. In particular, it is important to understand whether the neuropsychiatric components of posthospitalization syndrome for COVID-19 differ from those of other severe respiratory infections treated in the same settings.

Better understanding of the neuropsychiatric sequelae of severe COVID-19 and non–COVID-19 SARI is relevant to clinical practice, and both pandemic and postpandemic planning. Using electronic health record data for a population-representative cohort of more than 8 million individuals in England, this study aims to quantify the neuropsychiatric sequelae following discharge from COVID-19 hospitalization and to compare findings with patients surviving hospitalization due to non–COVID-19 SARI.

## Methods

This study was conducted in accordance with our protocol^10^ and is reported in accordance with the Strengthening the Reporting of Observational Studies in Epidemiology (STROBE) reporting guideline for observational studies. Protocol deviations are described in the eMethods in the Supplement. This study received approval from the QResearch Scientific Committee, which has ethical approval from the East Midlands-Derby Research Ethics Committee (reference 18/EM/0400, project reference OX79). Individual patient informed consent was not required because this study used anonymized patient records.

### Study Population and Data Sources

We used QResearch (version 45), a primary care database that has collected routine clinical data for more than 30 million individuals since 1989 registered to more than 1400 general practices in England using the EMIS software system. QResearch has individual-level linkage to Hospital Episode Statistics (HES), the Public Health England Second Generation Surveillance System database regarding SARS-CoV-2 reverse transcriptase–polymerase chain reaction testing, the Intensive Care National Audit and Research Centre Case Mix Program database, and the Office for National Statistics’ national mortality register.

This study cohort comprised adults alive and registered with a contributing general practice from January 24, 2020 (date of first COVID-19 case in England), until the data extract date (July 7, 2021). We extracted a temporally distinct historic cohort to compare incidence rates of post-SARI discharge neuropsychiatric sequelae with the remaining population, intending to statistically compare those in the prepandemic period with those in the contemporary pandemic period. This cohort identified adults aged 18 years and older entering the cohort from January 24, 2015, to January 23, 2020.

### Exposures and Outcomes

Exposures were being discharged alive from (surviving) a COVID-19–related hospital admission and being discharged alive from (surviving) an SARI-related hospital admission. The reference group was the remaining population. COVID-19–related hospital admissions were defined as those with a recorded *International Statistical Classification of Diseases and Related Health Problems, Tenth Revision (ICD-10)* code (U07.1 or U07.2) as the reason for admission to hospital (in the HES data set), or admission to hospital for any reason within 14 days of a positive SARS-CoV-2 reverse transcriptase–polymerase chain reaction test result. SARI-related hospital admissions were defined as those with *ICD-10* codes J09 to J22 as a reason for admission in the HES data set, or as the reason for admission to intensive care in the Intensive Care National Audit and Research Centre Case Mix Program data set.

Outcomes of interest comprised new-onset diagnoses of selected neuropsychiatric conditions or first prescriptions for selected medications occurring after hospital discharge (or otherwise occurring during follow-up in the general population). For each outcome, we excluded individuals with previously recorded records of the outcome of interest (eg, participants with previous records of anxiety were dropped from models for anxiety but eligible for inclusion in models for other end points if they did not have that also recorded before follow-up start date). We used Systematized Nomenclature of Medicine/Read codes in the primary care records, and *ICD-10* codes in the HES records to identify individuals formally diagnosed by a health care practitioner with anxiety disorder, depression, dementia, bipolar affective disorder, and psychotic disorder. Although the core QResearch data set is a primary care database, individual level linkage across primary and secondary/specialist care data permitted capture of recorded psychiatric diagnoses in either setting. Primary care records were used to identify prescriptions of relevant medications based on the British National Formulary: antidepressants, hypnotics/anxiolytics, and antipsychotics. These code lists are available on the QResearch website.^11^

We compared the risk of new-onset neuropsychiatric diagnoses or first prescriptions in the 12 months following discharge in people surviving a COVID-19–related or SARI-related hospital admission (including intensive care) to the risk in the remaining population. Follow-up time was calculated from the discharge date for those surviving a COVID-19–related or SARI-related hospital admission. Follow-up for the remaining population was from January 24, 2020. Patient follow-up was until the earliest of date of outcome of interest or censoring (left practice, died for any reason, or reached the end of follow-up alive). Follow-up was truncated to 1 year for analysis. In the historic cohort, SARI admissions, exposures, and outcomes had identical definitions as the main cohort. SARI admission survivors were followed up from date of discharge to date of new-onset diagnosis/first prescription. Follow-up was truncated at 1 year.

### Statistical Analysis

Crude and age-standardized incidence rates for each end point were calculated in contemporary and historic cohorts for initial comparisons. Flexible parametric survival models were used to estimate hazard ratios (HRs) with 95% CIs for each of the outcomes, using 4 *df* for the baseline survival function splines and clustered robust SEs to account for patient clustering within individual practices.

All final analysis models were adjusted for demographic and clinical factors identified from clinical/epidemiological understanding as summarized in the directed acyclic graphs in the protocol and shown in Table 1. Age was handled using restricted cubic splines with 4 knots. Ethnic group categorization was determined by event counts. Using self-reported ethnicity data and with reference to Office for National Statistics classifications, for the purposes of analysis we generated the following groups: Asian (South Asian, Chinese, and other Asian), Black (Black African, Black Caribbean, and other Black), White (British or other White), and other (including Arab, and multiple ethnicity) groups.^12^ Missing data were observed for ethnicity, smoking status, alcohol status, body mass index (BMI) and Townsend Deprivation Index quintile. Under the missing at random assumption, multiple imputation with chained equations was used to replace missing values.^13^ The imputation model included all covariates and outcomes. Five imputations were generated, with model coefficients and SEs pooled across imputations in accordance with Rubin’s rules.^14^

**Table 1.** Sociodemographic and Clinical Characteristics of the Primary Analysis Cohort

Characteristic	No. (%)		
	Reference population (N = 8 330 986)	Admission survivors	
		SARI (n = 16 679)	COVID-19 (n = 32 525)
Age, mean (SD), y	49.07 (18.40)	69.34 (18.70)	65.40 (18.42)
BMI, mean (SD)	26.83 (5.63)	27.66 (6.32)	29.38 (6.41)
Sex			
Female	4 154 145 (49.86)	8239 (49.40)	15 116 (46.48)
Male	4 176 841 (50.14)	8440 (50.60)	17 409 (53.52)
Region in England			
East Midlands	204 681 (2.46)	358 (2.15)	486 (1.49)
East of England	323 725 (3.89)	582 (3.49)	1006 (3.09)
London	2 021 953 (24.27)	2646 (15.86)	9236 (28.40)
North East	204 733 (2.46)	373 (2.24)	785 (2.41)
North West	1 565 669 (18.79)	3891 (23.33)	7548 (23.21)
South Central	1 082 614 (13.00)	2332 (13.98)	3353 (10.31)
South East	908 825 (10.91)	1970 (11.81)	3516 (10.81)
South West	854 863 (10.26)	1862 (11.16)	1909 (5.87)
West Midlands	839 454 (10.08)	1945 (11.66)	3653 (11.23)
Yorkshire and Humber	324 469 (3.89)	720 (4.32)	1033 (3.18)
Race and ethnicity^a^			
Asian	729 638 (8.76)	841 (5.04)	4388 (13.49)
Black	282 535 (3.39)	362 (2.17)	1896 (5.83)
White	5 350 291 (64.22)	12 368 (74.15)	20 278 (62.35)
Other^b^	287 317 (3.45)	294 (1.76)	1262 (3.88)
Not recorded	1 681 205 (20.18)	2814 (16.87)	4701 (14.34)
Townsend deprivation score 5th			
1	2 071 212 (24.86)	4066 (24.38)	6362 (19.56)
2	1 822 234 (21.87)	3777 (22.65)	6435 (19.78)
3	1 625 875 (19.52)	3541 (21.23)	6792 (20.88)
4	1 440 868 (17.30)	3030 (18.17)	6645 (20.43)
5	1 332 635 (16.00)	2221 (13.32)	6182 (19.01)
Not recorded	38 162 (0.46)	44 (0.26)	109 (0.34)
Smoking status (cigarettes/d)			
None	4 787 860 (57.47)	7610 (45.63)	18 683 (57.44)
Former	1 783 763 (21.41)	5895 (35.34)	10 923 (33.58)
Light smoker (1-9)	1 095 069 (13.14)	2210 (13.25)	2079 (6.39)
Moderate smoker (10-19)	214 696 (2.58)	511 (3.06)	393 (1.21)
Heavy smoker (≥20)	98 084 (1.18)	334 (2.00)	210 (0.65)
Not recorded	351 514 (4.22)	119 (0.71)	237 (0.73)
Alcohol intake, u/d			
None	4 298 748 (51.60)	108 80 (65.23)	22 143 (68.08)
Trivial (<1)	1 252 268 (15.03)	2347 (14.07)	4393 (13.51)
Light (1-2)	624 708 (7.50)	1042 (6.25)	2007 (6.17)
Moderate (3-6)	486 201 (5.84)	1023 (6.13)	1555 (4.78)
Heavy (7-9)	38 988 (0.47)	134 (0.80)	163 (0.50)
Very heavy (>9)	35 584 (0.43)	94 (0.56)	103 (0.32)
Not recorded	1594 489 (19.14)	1159 (6.95)	2161 (6.64)
Coronary artery disease	305 183 (3.66)	3052 (18.30)	4910 (15.10)
Hypertension	1 484 600 (17.82)	8077 (48.43)	15 033 (46.22)
Atrial fibrillation	208 182 (2.50)	2722 (16.32)	3714 (11.42)
Congestive cardiac failure	99 349 (1.19)	1806 (10.83)	2582 (7.94)
Stroke	183 707 (2.21)	2080 (12.47)	3269 (10.05)
Peripheral vascular disease	63 011 (0.76)	892 (5.35)	1208 (3.71)
Venous thromboembolism	152 104 (1.83)	1581 (9.48)	2577 (7.92)
Chronic obstructive pulmonary disease	198 242 (2.38)	2688 (16.12)	3134 (9.64)
Asthma	112 3517 (13.49)	3209 (19.24)	5613 (17.26)
Bronchiectasis	43 132 (0.52)	622 (3.73)	662 (2.04)
Epilepsy	112 568 (1.35)	615 (3.69)	891 (2.74)
Multiple sclerosis	21 018 (0.25)	105 (0.63)	179 (0.55)
Osteoarthritis	939 125 (11.27)	5243 (31.43)	9393 (28.88)
Gastrointestinal cancer	50 442 (0.61)	567 (3.40)	699 (2.15)
Urological cancer	97 380 (1.17)	835 (5.01)	1230 (3.78)
Gynecological cancer	24 648 (0.30)	130 (0.78)	217 (0.67)
Rarer pulmonary disorders^c^	18 945 (0.23)	392 (2.35)	454 (1.40)
Rheumatoid arthritis/systemic lupus erythematosus	75 851 (0.91)	564 (3.38)	816 (2.51)
Sickle cell disease/severe combined immunodeficiency	6605 (0.08)	73 (0.44)	94 (0.29)
Diabetes			
Type 1	46 412 (0.56)	253 (1.52)	510 (1.57)
Type 2	57 4216 (6.89)	3626 (21.74)	8753 (26.91)
Severe head injury	46 126 (0.55)	101 (0.61)	153 (0.47)
Hypothyroidism	370 369 (4.45)	1711 (10.26)	2834 (8.71)
Learning disability	131 275 (1.58)	595 (3.57)	1017 (3.13)
Chronic kidney disease	351 982 (4.22)	3586 (21.50)	6123 (18.83)
Cancer			
Breast	102 867 (1.23)	554 (3.32)	754 (2.32)
Blood	43 651 (0.52)	582 (3.49)	764 (2.35)
Chronic liver disease or chronic pancreatitis	64 483 (0.77)	576 (3.45)	834 (2.56)
Previous fracture	333 969 (4.01)	2086 (12.51)	2896 (8.90)
Anticoagulation therapy	246 033 (2.95)	2215 (13.28)	2255 (6.93)
Steroid therapy	784 219 (9.41)	3815 (22.87)	4868 (14.97)
HRT use			
Estrogen only	193 423 (2.32)	404 (2.42)	618 (1.90)
Progestogen only	350 461 (4.21)	477 (2.86)	1074 (3.30)
Combined	95 469 (1.15)	213 (1.28)	350 (1.08)
Anticonvulsant therapy	484 521 (5.82)	2934 (17.59)	3864 (11.88)
Bisphosphonates	132 381 (1.59)	1003 (6.01)	1026 (3.15)
Leukotriene antagonist	1 360 502 (16.33)	4792 (28.73)	6675 (20.52)
ACE inhibitor	788 654 (9.47)	2693 (16.15)	4429 (13.62)
NSAIDs	1 810 629 (21.73)	3005 (18.02)	5572 (17.13)
Aspirin	506 129 (6.08)	2382 (14.28)	3579 (11.00)
Cytotoxic immunotherapy	22 427 (0.27)	139 (0.83)	189 (0.58)
Statins	1 280 862 (15.37)	5114 (30.66)	8182 (25.16)

Abbreviations: ACE, angiotensin converting enzyme; BMI, body mass index (calculated as weight in kilograms divided by height in meters squared); HRT, hormone replacement therapy; NSAIDS, nonsteroidal anti-inflammatory drugs; u, alcohol units.

^a^ Race and ethnic group categorization was determined by event counts. Using self-reported ethnicity data and with reference to Office for National Statistics classifications, for the purposes of analysis we generated the following groups: Asian (South Asian, Chinese, and other Asian), Black (Black African, Black Caribbean, and other Black), White (British or other White), and other (including Arab, and multiple ethnicity) groups.

^b^ Other ethnic group refers to Office for National Statistics categories such as Arab and multiple ethnicity.

^c^ The group rare pulmonary diseases comprises cystic fibrosis, alveolitis, and pulmonary fibrosis.

For a direct comparison of exposures, we repeated these analyses restricting the data set to those that survived either a COVID-19–related or SARI-related hospital admission. Herein, HRs with 95% CIs were calculated to estimate the hazards of neuropsychiatric sequelae in survivors of COVID-19 hospitalization, with survivors of SARI hospitalization as the reference group.

### Sensitivity Analyses

With 1-year follow-up for modeling in the context of 1.5-year study period, the time frames in which different exposure groups contributed follow-up varies due to follow-up starting postdischarge for the exposed groups, but at the index date set at January 24, 2020, for the general population. There is also potential for baseline diagnostic rate variation during the pandemic, especially considering several lockdowns in England. Therefore, we repeated the modeling steps, but those in the reference population group were randomly assigned a cohort start date in between the first discharge date and the latest discharge date identified in the exposed groups. We also adjusted for the calendar month of this updated study start date (ie, discharge date if a survivor of SARI/COVID-19 hospitalization or the newly assigned date if in the reference group). We repeated our analyses excluding those that did not have COVID-19 as the primary reason for their hospital admission or admission to the intensive care unit (eg, those with a recent positive test, but not admitted primarily due to COVID-19) from the COVID-19 exposure group.

We also compared the risks of our end points of interest after SARI/COVID-19 with individuals surviving an admission with acute myocardial infarction (*ICD-10* code group I21) during the same period (January 24, 2020, to July 7, 2021). Those with both acute myocardial infarction admissions and SARI-related or COVID-19–related hospitalizations during the study period were excluded; maximally adjusted HRs were estimated as described above; the acute myocardial infarction group was the reference group.

Statistical significance was based on whether 95% CIs for maximally adjusted HRs crossed 1. All data manipulation and analyses were conducted using Stata, version 17 (StataCorp LLC).

## Results

### Study Cohorts

There were 8 380 190 people included in the final contemporary study cohort and 12 162 155 included in the prepandemic cohort (SARI or remaining population). In the former, 7 058 199 reached 1 year follow-up (84.22%).

### Primary Analyses

This cohort study comprised data from 8.38 million adults (4.18 million women, 4.20 million men; mean [SD] age 49.18 [18.45] years). Of the contemporary cohort, 519 134 had at least 1 positive SARS-CoV-2 reverse transcriptase-polymerase chain reaction test result recorded, and there were 50 798 hospital admissions with COVID-19. At the time of data extract, 16 679 (0.02%) had survived (reached discharge after) a hospital admission with SARI, and 32 525 (0.04%) had survived (reached discharge after) a hospital admission with COVID-19. Due to the low event counts for some endpoints, we did not undertake a subanalysis of survivors of intensive care unit admissions.

Table 1 summarizes the demographic and clinical characteristics of the final cohort for the analyses, derived following the steps outlined in the flowchart in the eFigure in the Supplement. Event counts occurring during follow-up are presented in Table 2. Crude and age-standardized incidence rates of neuropsychiatric sequelae in the historic and contemporary cohorts are summarized in eTable 1 in the Supplement. Due to different risks of several outcomes of interest in the historic and contemporary cohorts regarding survivors of SARI hospitalization, we did not undertake formal modeling comparing patients in the historic cohort with those in the primary cohort.

**Table 2.** Event Counts in the Contemporary Study Cohort, Stratified by Exposure

Outcome^a^	No. (%)		
	Reference population	Admission survivors	
		SARI	COVID-19
New-onset diagnosis			
Anxiety	72 568 (0.95)	137 (1.04)	179 (0.74)
Dementia	14 447 (0.18)	156 (1.13)	126 (0.50)
Psychotic disorder	1915 (0.02)	<10	10 (0.04)
Depression	4519 (0.06)	19 (0.15)	12 (0.05)
Bipolar affective disorder	4159 (0.05)	12 (0.08)	12 (0.04)
New prescription			
Antidepressant	115 338 (2.10)	263 (4.12)	378 (2.85)
Hypnotic/anxiolytic	64 126 (0.99)	294 (3.44)	362 (2.11)
Antipsychotic	27 790 (0.34)	529 (3.90)	412 (1.64)

Abbreviation: SARI, severe acute respiratory infections.

^a^ The numbers of new-onset diagnoses or prescriptions refer to those occurring within 1 year of follow-up. Due to the removal of previously recorded diagnosis or prior prescriptions for each statistical model, the denominators may vary for each end point of interest.

**Figure 1.**
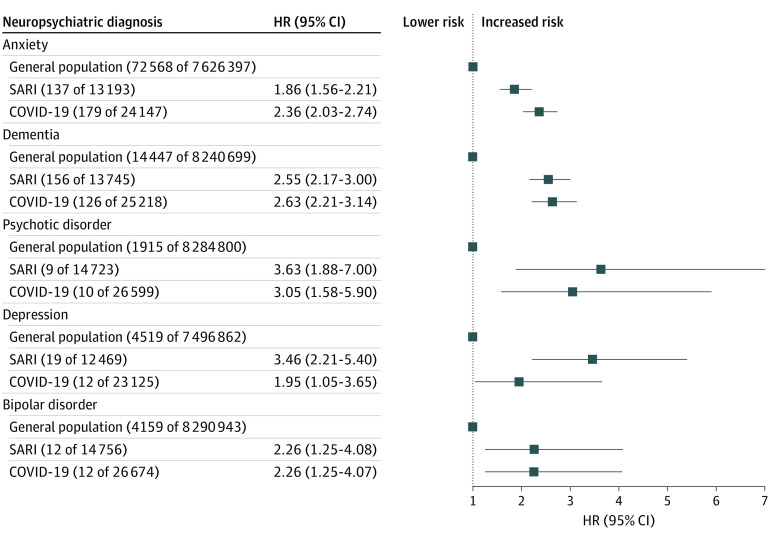
First Recorded New-Onset Neuropsychiatric Diagnosis After Hospital Discharge in the Contemporary Cohort Numbers in parentheses following general population, SARI, and COVID-19 refer to number of events and denominator. Inclusion was determined on a per-analysis basis, and therefore, the denominator counts may vary. HR indicates hazard ratio; SARI, severe acute respiratory infections.

**Figure 2.**
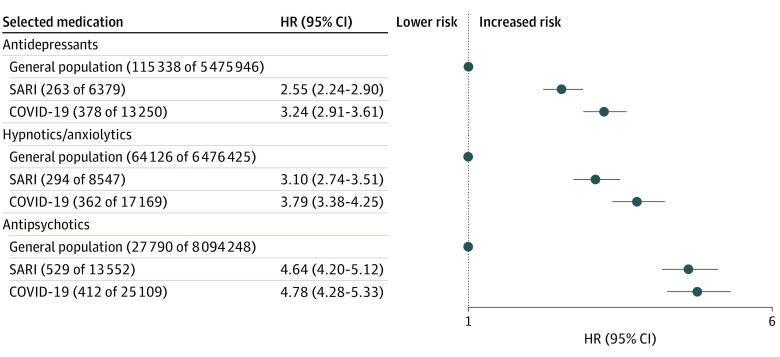
First Recorded Neuropsychiatric Prescription After Hospital Discharge in the Contemporary Cohort Numbers in parentheses following general population, SARI, and COVID-19 refer to number of events and denominator. Inclusion was performed on a per-analysis basis, and therefore, the denominator counts may vary. HR indicates hazard ratio; SARI, severe acute respiratory infections.

In the primary study cohort, patients surviving SARI or COVID-19–related admissions had higher risks of all new-onset neuropsychiatric diagnoses within 12 months compared with the remaining population, and the adjusted HRs for both exposures were generally similar (Figure 1). Compared with the wider population, the maximally adjusted HR for anxiety disorder for the SARI group was 1.86 (95% CI, 1.56-2.21) and for the COVID-19 group was 2.36 (95% CI, 2.03-2.74), and the maximally adjusted HR for dementia in the SARI group was 2.55 (95% CI, 2.17-3.00) and for the COVID-19 group was 2.63 (95% CI, 2.21-3.14). Survivors of SARI and COVID-19 hospital admission also had increased risks of commencing treatment with antidepressants (adjusted HR for survivors of SARI, 2.55 [95% CI, 2.24-2.90] and for survivors of COVID-19, 3.24 [95% CI, 2.91-3.61]), treatment with hypnotics/anxiolytics (adjusted HR for survivors of SARI, 3.10 [95% CI, 2.74-3.51] and for survivors of COVID-19, 3.79 [95% CI, 3.38-4.25]), and treatment with antipsychotic medications (adjusted HR for survivors of SARI, 4.64 [95% CI, 4.20-5.12], and for survivors of COVID-19, 4.78 [95% CI, 4.28-5.33]) (Figure 2).

When the analysis was restricted to survivors of SARI hospitalization as the reference group and survivors of COVID-19 hospitalization as the comparator, no significant differences were observed in the postdischarge rates of new-onset anxiety disorder (adjusted HR, 1.00; 95% CI, 0.79-1.27), dementia (adjusted HR, 0.88; 95% CI, 0.69-1.13), depression (adjusted HR, 0.63; 95% CI, 0.25-1.58), or bipolar affective disorder (adjusted HR, 0.74; 95% CI, 0.32-1.69). Further, the 2 groups did not differ in terms of their postdischarge risks of antidepressant medication prescription (adjusted HR, 1.07; 95% CI, 0.90-1.27) or hypnotic/anxiolytic medication prescription (adjusted HR, 0.95; 95% CI, 0.80-1.24). Those surviving COVID-19–related hospitalization did have a 20% lower risk of receiving their first-ever antipsychotic medication prescription compared with those surviving an SARI-related admission (adjusted HR, 0.80; 95% CI, 0.69-0.92). eTable 2 in the Supplement summarizes unadjusted, age- and sex-adjusted, and maximally adjusted HRs.

### Sensitivity Analyses

The results after accounting for calendar time were similar to the primary analyses for both diagnoses and prescriptions (eTable 3 in the Supplement).

Of the COVID-19–related hospitalization subset, 20 604 individuals (66.35%) had COVID-19 recorded as the primary reason for admission. The remaining 33.65% were admitted within 14 days of a positive SARS-CoV-2 test result, but COVID-19 was not specifically coded as the primary reason for admission. Excluding the latter, we observed HRs consistent those found in the primary analysis (eTable 4 in the Supplement).

Using individuals surviving admission after acute myocardial infarction as the reference group (n = 10 630), risks associated with COVID-19 and SARI admissions varied (Table 3). There were lower risks of new-onset anxiety disorder in the SARI group (adjusted HR, 0.66; 95% CI, 0.49-0.88) and COVID-19 group (adjusted HR, 0.62; 95% CI, 0.46-0.83) but increased risks of new-onset dementia (adjusted HR in the SARI group, 2.24 [95% CI, 1.53-3.27], and in the COVID-19 group, 1.92 [95% CI, 1.28-2.87]), new prescriptions of antidepressants (adjusted HR in the SARI group, 1.46; 95% CI, 1.16-1.85, and in the COVID-19 group, 1.55 [95% CI, 1.24-1.94]), and antipsychotics (adjusted HR in the SARI group, 2.48 [95% CI, 1.96-3.15], and in the COVID-19 group, 2.00 [95% CI, 1.58-2.54]) (Table 3).

**Table 3.** Comparison of Risks of New-Onset Diagnoses First Prescriptions, Associated With COVID-19 and SARI Admissions

Outcome^a^	Maximally adjusted hazard ratio (95% CI)
**New-onset anxiety diagnosis**	
Acute myocardial infarction	1 [Reference]
SARI	0.66 (0.49-0.88)
COVID-19	0.62 (0.46-0.83)
**New-onset dementia diagnosis**	
Acute myocardial infarction	1 [Reference]
SARI	2.24 (1.53-3.27)
COVID-19	1.92 (1.28-2.87)
**New-onset depression diagnosis**	
Acute myocardial infarction	1 [Reference]
SARI	1.16 (0.48-2.81)
COVID-19	0.68 (0.23-2.07)
**New antidepressant prescription**	
Acute myocardial infarction	1 [Reference]
SARI	1.46 (1.16-1.85)
COVID-19	1.55 (1.24-1.94)
**New hypnotic/anxiolytic prescription**	
Acute myocardial infarction	1 [Reference]
SARI	1.09 (0.88-1.35)
COVID-19	1.04 (0.83-1.28)
**New antipsychotic prescription**	
Acute myocardial infarction	1 [Reference]
SARI	2.48 (1.96-3.15)
COVID-19	2.00 (1.58-2.54)

Abbreviation: SARI, severe acute respiratory infections.

^a^ These are compared with the risks seen in survivors of hospitalization for acute myocardial infarction during the study period (reference group; January 24, 2020, to July 7, 2021).

## Discussion

In this study using a population-level cohort comprising linked electronic primary and secondary health care data sets for more than 8 million adults, we estimated that the neuropsychiatric ramifications of severe COVID-19 infection were similar to those of other SARIs. Individuals surviving severe COVID-19 infection and other SARIs were at significantly increased risk of receiving a neuropsychiatric illness diagnosis and of being prescribed antidepressant, hypnotic/anxiolytic, or antipsychotic medications in the first year after discharge compared with the wider population. Apart from a reduced risk of being prescribed antipsychotic medication, the differences between risks post–COVID-19 hospitalization and post-SARI hospitalization were not significant. Although the relative risks of these outcomes in survivors of COVID-19 and SARI hospitalizations are significantly higher than for the remaining population, the absolute risks for some formally diagnosed neuropsychiatric sequelae are low (eTable 1 in the Supplement).

Postacute outcomes associated with severe respiratory infections span physical, cognitive, and psychological domains^5,15^; could be influenced by physiological perturbations, deconditioning, and other stressors^16^; and are recognized under umbrella diagnostic entities such as the postintensive care syndrome,^15,17^ posthospitalization syndrome,^16^ and in some cases postviral syndrome.^18^ Although post–COVID-19 syndrome^19^ is of legitimate topical interest in the current context of uncertainty regarding optimal support for survivors of COVID-19, our results suggest that from the perspective of formally diagnosed or treated neuropsychiatric complications, severe COVID-19 does not predicate markedly different morbidity rates than other forms of SARI.

Using the TriNetX data set, the Taquet and colleagues^20^ propensity score–matched study reported that the relative risks of several neuropsychiatric conditions after any SARS-CoV-2 infection (regardless of severity) exceed those for any respiratory tract infection (including influenza) at 6 months of follow-up. However, comparison with influenza may be problematic because of its atypically low incidence during the COVID-19 pandemic and its markedly different risk profile.^8,21^ Comparisons with recorded diagnosis of any acute respiratory infection may also be complicated owing to different severity profiles. In contrast, we compared outcomes following severe infection with COVID-19 (defined as requiring hospitalization) with severe infection with a broader set of SARIs. We saw similar rates of neuropsychiatric outcomes, both substantially higher than in the general population. Our results suggest that increased rates of the neuropsychiatric outcomes may be associated with the severity of respiratory disease rather than the underlying organism. Notably, the Taquet et al study^20^ also appeared to have higher incidence of neuropsychiatric diagnoses than we encountered in our English cohort, which may reflect different diagnostic practices and/or disruption thereto in the US and England.

### Strengths and Limitations

Strengths of our study include using a large cohort that is broadly representative of the English general population, and the use of multiple validated electronic health care data sets linked at the individual level for accurate ascertainment of exposures (including lifestyle factors), outcomes, and relevant confounders. In contrast to previous studies^9,20^ analyzing data from during the COVID-19 pandemic, we also explored background risk factors in the general population during and prior to the pandemic. Our source data sets minimize selection and recall bias because they use health record data from a large representative population. Our comparison with SARI enabled more informative benchmarking than some other studies using a less severe infection.^7,20^

This study also has limitations, including the fact that several of the neuropsychiatric conditions examined typically first manifest in younger age (in the context of an analysis subcohort mean age >60 years). The use of routinely collected health care data means that not all outcomes and exposures are formally adjudicated, as the database is dependent on coding by individual practitioners. There may also be information or recording bias due to disruption in patient attendance at general practices during the study period, reduced service availability, or changes in help-seeking behaviors such as avoidance of hospital-based or outpatient care by some individuals. In contrast, increased attention to post–COVID-19 syndrome may risk surveillance bias, increasing diagnosis rates in the COVID-19 group. Optimal comparison group selection therefore, may be complex in the setting of multifaceted indirect effects of the pandemic in terms of health care services. Prepandemic rates of new-onset posthospitalization neuropsychiatric diagnoses appeared to differ from that seen in the contemporary cohort, precluding temporal comparisons, and patients admitted to hospital with non–COVID-19 SARI during the COVID-19 pandemic may be different from those admitted prepandemic. However, we believe that our contemporaneous design is the most robust with regard to indirect effects of the pandemic and offered a meaningful comparison. It could be informative to compare neuropsychiatric complications and potential mediators in individuals surviving admissions for other conditions beyond acute myocardial infarction in future studies.

## Conclusions

In this cohort study, SARI were found to be associated with significant postacute neuropsychiatric morbidity, for which COVID-19 is not distinctly different. These results may help refine our understanding of the postsevere COVID-19 phenotype and may inform postdischarge support for patients requiring hospital-based and intensive care for SARI regardless of causative pathogen.

## Notes

Supplement. **eMethods.** Deviations From Study Protocol **eFigure 1.** Flowchart Summarizing Study Cohort Derivation **eTable 1.** Incidence Rates for Neuropsychiatric Sequelae in Historic and Contemporary Cohorts **eTable 2.** Unadjusted, Age/Sex-Adjusted, and Maximally Adjusted Hazard Ratios for Primary Analysis **eTable 3.** Sensitivity Analysis Results (Calendar and Follow-up Time) **eTable 4.** Sensitivity Analysis Results (Restricting to Those With COVID-19 as Primary Reason for Admission on Hospitalization Record)

## References

[1] Johns Hopkins Coronavirus Resource Center. COVID-19 map. Accessed March 1, 2022. https://coronavirus.jhu.edu/map.html.

[2] Crook H, Raza S, Nowell J, Young M, Edison P. Long covid-mechanisms, risk factors, and management. BMJ. 2021;374(1648):n1648. doi:10.1136/bmj.n1648 34312178

[3] Nalbandian A, Sehgal K, Gupta A, . Post-acute COVID-19 syndrome. Nat Med. 2021;27(4):601-615. doi:10.1038/s41591-021-01283-z 33753937 PMC8893149

[4] Hatch R, Young D, Barber VS, Griffiths J, Harrison DA, Watkinson PJ. Anxiety, depression and post-traumatic stress disorder management after critical illness: a UK multi-centre prospective cohort study. Crit Care. 2020;24(1):633. doi:10.1186/s13054-020-03354-y 33138832 PMC7607621

[5] Pandharipande PP, Girard TD, Ely EW. Long-term cognitive impairment after critical illness. N Engl J Med. 2014;370(2):185-186. 24401069 10.1056/NEJMc1313886

[6] Rogers JP, Chesney E, Oliver D, . Psychiatric and neuropsychiatric presentations associated with severe coronavirus infections: a systematic review and meta-analysis with comparison to the COVID-19 pandemic. Lancet Psychiatry. 2020;7(7):611-627. doi:10.1016/S2215-0366(20)30203-0 32437679 PMC7234781

[7] Taquet M, Luciano S, Geddes JR, Harrison PJ. Bidirectional associations between COVID-19 and psychiatric disorder: retrospective cohort studies of 62354 COVID-19 cases in the USA. Lancet Psychiatry. 2021;8(2):130-140. doi:10.1016/S2215-0366(20)30462-4 33181098 PMC7820108

[8] Cowling BJ, Ali ST, Ng TWY, . Impact assessment of non-pharmaceutical interventions against coronavirus disease 2019 and influenza in Hong Kong: an observational study. Lancet Public Health. 2020;5(5):e279-e288. doi:10.1016/S2468-2667(20)30090-6 32311320 PMC7164922

[9] Evans RA, McAuley H, Harrison EM, ; PHOSP-COVID Collaborative Group. Physical, cognitive, and mental health impacts of COVID-19 after hospitalisation (PHOSP-COVID): a UK multicentre, prospective cohort study. Lancet Respir Med. 2021;9(11):1275-1287. doi:10.1016/S2213-2600(21)00383-0 34627560 PMC8497028

[10] QResearch-ICNARC Collaboration. Investigating neuropsychological disease and treatments before and after severe COVID-19 disease. Published July 2021. Accessed April 6, 2022. https://www.qresearch.org/media/1338/ox79-qresearch-icnarc-covid-19-psychological-outcomes.pdf

[11] QResearch. QCode Group Library. Accessed April 6, 2022. https://www.qresearch.org/qcode-group-library/

[12] GOV.UK. Ethnicity facts and figures: list of ethnic groups. Accessed April 15, 2022. https://www.ethnicity-facts-figures.service.gov.uk/style-guide/ethnic-groups

[13] Sterne JA, White IR, Carlin JB, . Multiple imputation for missing data in epidemiological and clinical research: potential and pitfalls. BMJ. 2009;338:b2393. doi:10.1136/bmj.b2393 19564179 PMC2714692

[14] Rubin D. Multiple Imputation for Nonresponse in Surveys. Wiley; 1987. doi:10.1002/9780470316696

[15] Rousseau AF, Prescott HC, Brett SJ, . Long-term outcomes after critical illness: recent insights. Crit Care. 2021;25(1):108. doi:10.1186/s13054-021-03535-3 33731201 PMC7968190

[16] Krumholz HM. Post-hospital syndrome–an acquired, transient condition of generalized risk. N Engl J Med. 2013;368(2):100-102. doi:10.1056/NEJMp1212324 23301730 PMC3688067

[17] Rousseau AF, Minguet P, Colson C, . Post-intensive care syndrome after a critical COVID-19: cohort study from a Belgian follow-up clinic. Ann Intensive Care. 2021;11(1):118. doi:10.1186/s13613-021-00910-9 34324073 PMC8319705

[18] Hickie I, Davenport T, Wakefield D, ; Dubbo Infection Outcomes Study Group. Post-infective and chronic fatigue syndromes precipitated by viral and non-viral pathogens: prospective cohort study. BMJ. 2006;333(7568):575. doi:10.1136/bmj.38933.585764.AE 16950834 PMC1569956

[19] Greenhalgh T, Knight M, A’Court C, Buxton M, Husain L. Management of post-acute covid-19 in primary care. BMJ. 2020;370:m3026. doi:10.1136/bmj.m3026 32784198

[20] Taquet M, Geddes JR, Husain M, Luciano S, Harrison PJ. 6-month neurological and psychiatric outcomes in 236 379 survivors of COVID-19: a retrospective cohort study using electronic health records. Lancet Psychiatry. 2021;8(5):416-427. doi:10.1016/S2215-0366(21)00084-5 33836148 PMC8023694

[21] Rogers JP, David AS. A longer look at COVID-19 and neuropsychiatric outcomes. Lancet Psychiatry. 2021;8(5):351-352. doi:10.1016/S2215-0366(21)00120-6 33836149 PMC8023693

