# Supplementary material for: Neuropsychiatric Ramifications of Severe COVID-19 and Other Severe Acute Respiratory Infections
Source: JAMA Psychiatry. 2022 May 11;79(7):690–8. doi: 10.1001/jamapsychiatry.2022.1067 (PMC9096686; doi:10.1001/jamapsychiatry.2022.1067)
Supplement: Supplement. — eMethods. Deviations From Study Protocol eFigure 1. Flowchart Summarizing Study Cohort Derivation eTable 1. Incidence Rates for Neuropsychiatric Sequelae in Historic and Contemporary Cohorts eTable 2. Unadjusted, Age/Sex-Adjusted, and Maximally Adjusted Hazard Ratios for Primary Analysis eTable 3. Sensitivity Analysis Results (Calendar and Follow-up Time) eTable 4. Sensitivity Analysis Results (Restricting to Those With COVID-19 as Primary Reason for Admission on Hospitalization Record) [file jamapsychiatry-e221067-s001.pdf]

Supplemental Online Content

Clift AK, Ranger TA, Patone M, et al. Neuropsychiatric ramifications of severe COVID-19 and other severe acute respiratory infections. *JAMA Psychiatry*. Published online May 11, 2022. doi:10.1001/jamapsychiatry.2022.1067

eMethods. Deviations From Study Protocol

eFigure. Flowchart Summarising Study Cohort Derivation

eTable 1. Incidence Rates for Neuropsychiatric Sequelae in Historic and Contemporary Cohorts

eTable 2. Unadjusted, Age/Sex-Adjusted, and Maximally Adjusted Hazard Ratios for Primary Analysis

eTable 3. Sensitivity Analysis Results (Calendar and Follow-up Time)

eTable 4. Sensitivity Analysis Results (Restricting to Those With COVID-19 as Primary Reason for Admission on Hospitalization Record)

This supplemental material has been provided by the authors to give readers additional information about their work.

© 2022 Clift AK et al. *JAMA Psychiatry*.

eMethods. *Deviations from the protocol*

In the primary analysis cohort, we added a comparison of post-COVID-19 and post-SARI incidence to that in the general population, whereas only a direct comparison between the two diseases was initially planned. We believe the new analysis is more informative and relevant, but also present the results obtained using the initial analysis plan in the main paper. Additional outcomes were explored beyond those listed in the cohort based on clinical relevance, these were: Bipolar disorder (ICD-10 codes F30-31 and corresponding Read codes), Depression (F06.3, F32-F33, F34.1, F41.2, F44.8, F92.0 and corresponding Read codes) and Schizophrenia (F06.2, F20, F23.0-F23.2 and corresponding Read codes). Bipolar disorder is included in the broader category of mood disorders, schizophrenia is included in the category psychotic disorders and anti-depressant use is included in the hypnotics and anxiolytics category, nevertheless they were highlighted for their particular clinical importance and we have been careful not to over-interpret the results beyond those of the broader categories. We did not have sufficient event counts for incident schizophrenia diagnoses for a formal analysis.

A frailty term was initially proposed for the proportional hazards models to account for the clustered nature of the primary care data, but the use of Royston-Parmar models (Stata package stpm2) precluded this. Instead, we calculated robust standard errors clustered by general practice to account for clustering therein. We also proposed to combine the pre-pandemic and contemporary cohorts as part of the sequelae analysis in order to conduct competing risk analysis and calculate propensity scores of the likelihood of an event. The intention behind combining these cohorts was to account for potentially low event counts, but this proved unnecessary. Furthermore, combining them would have necessitated exclusion of patients who experienced an event in both cohorts, and may have been problematic due to changes in baseline characteristics. We did not feel a reasonable assumption could be made to exclude one event or the other. Given the sufficiency of event numbers and the lack of an acceptable assumption we abandoned this sub-analysis.

The sensitivity analysis assigning a random 'start date' to members of the reference group that were not admitted to hospital was not initially planned in the protocol. The sensitivity analysis excluding those individuals in the COVID-19-hospitalization survivor set that did not have COVID-19 recorded as the primary reason for admission (e.g. those admitted with a positive test within the preceding 14 days, but not as primary reason for admission) was in response to peer review. The post-hoc analytic comparisons with acute myocardial infarction patients reaching hospital discharge were also added in response to peer review.

© 2022 Clift AK et al. *JAMA Psychiatry*.

eFigure. Flowchart demonstrating derivation of the final study cohort. SARI = severe acute respiratory infection.

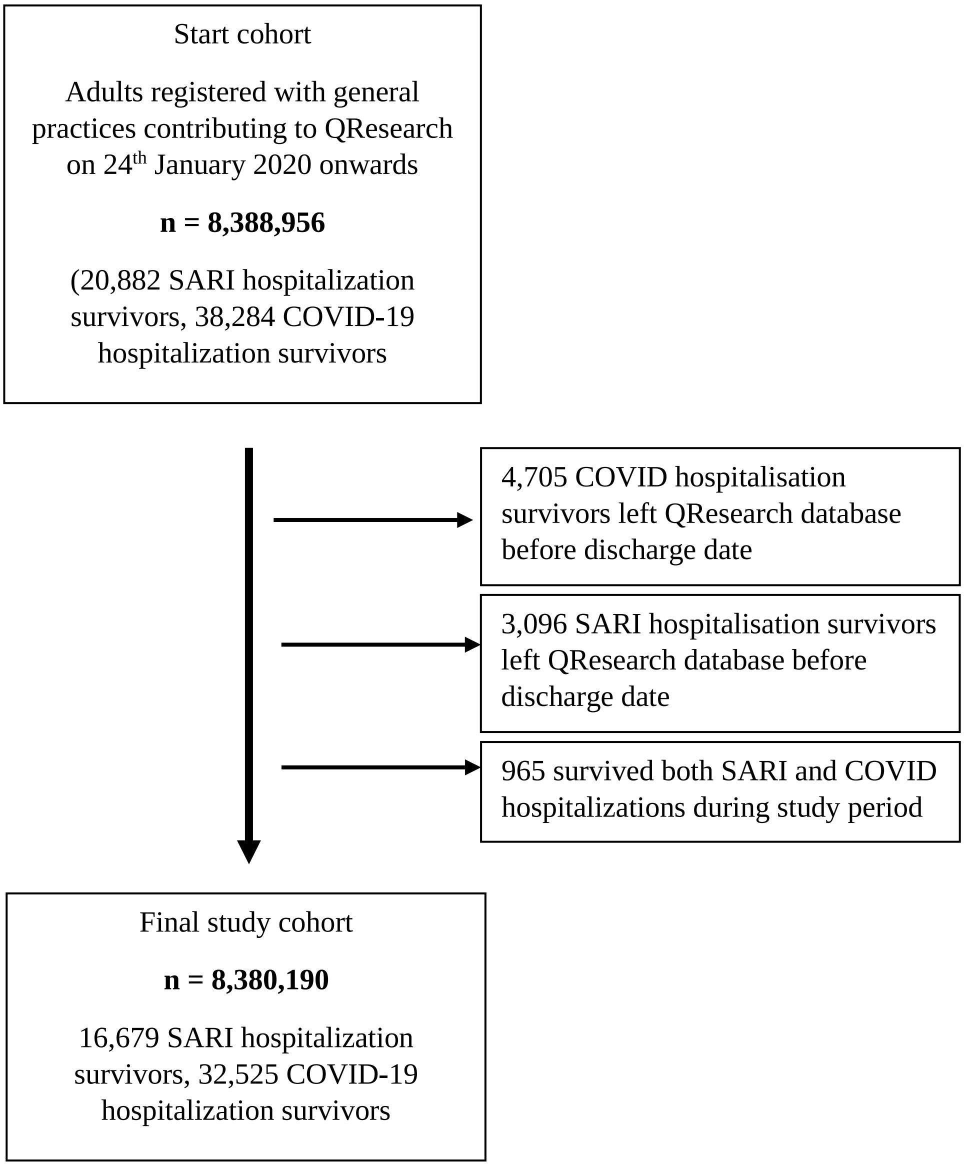

© 2022 Clift AK et al. *JAMA Psychiatry*.

			Historic cohort	Contemporary cohort		
			SARI hospitalization survivors n=212,935	SARI hospitalization survivors n=16,679	COVID hospitalization survivors n=32,525	Reference population n=8,330,986
**Crude incidence rate (per 100,000)**	Anxiety		414.56 (386.92 to 444.17)	1897.79 (1605.19 to 2243.73)	1975.36 (1706.18 to 2287.01)	992.95 (985.75 to 1000.20)
	Dementia		1555.48 (1499.86 to 1613.17)	2023.76 (1729.85 to 2367.06)	1310.28 (1100.36 to 1560.26)	181.90 (178.95 to 184.89)
	Mood disorder		370.50 (344.35 to 398.64)	478.16 (343.31 to 665.96)	357.23 (253.97 to 502.49)	141.55 (138.89 to 144.26)
	Psychosis		39.82 (31.93 to 49.64)	110.83 (57.67 to 213.01)	100.18 (53.90 to 186.19)	23.99 (22.94 to 25.09)
	Bipolar		19.62 (14.34 to 26.86)	147.46 (83.74 to 259.65)	119.85 (68.06 to 211.03)	52.08 (50.52 to 53.69)
	Depression		160.83 (142.99 to 180.90)	278.18 (177.44 to 436.12)	138.52 (78.67 to 243.91)	62.60 (60.80 to 64.45)
	Antidepressant use		1267.33 (1199.82 to 1338.64)	7592.52 (6728.20 to 8567.88)	7639.98 (6907.34 to 8450.32)	2213.37 (2200.64 to 2226.18)
	Hypnotic/anxiolytic use		1098.12 (1042.21 to 1157.02)	6274.21 (5596.49 to 7033.99)	5665.60 (5111.02 to 6280.35)	1034.13 (1026.16 to 1042.17)
	Anti-psychotic use		1128.54 (1081.15 to 1178.01)	7082.70 (6504.15 to 7712.72)	4378.99 (3975.93 to 4822.93)	356.51 (352.34 to 360.72)
**Age- and sex-standardized incidence rates (per 100,000)**	Anxiety	*F*	533.74 (489.45 to 578.04)	2150.85 (1677.04 to 2624.67)	3954.52 (3264.28 to 4644.76)	1395.57 (1383.30 to 1407.84)
		M	272.56 (238.44 to 306.68)	1649.76 (1229.63 to 2069.9)	1424.81 (1044.61 to 1807.06)	614.37 (606.55 to 622.20)
	Dementia	*F*	1666.80 (1591.61 to 1744.40)	2156.33 (1712.00 to 2600.66)	1892.63 (1447.17 to 2338.09)	187.94 (183.96 to 191.92)
		M	1355.19 (1275.47 to 1434.92)	1849.85 (1414.60 to 2285.11)	1603.49 (1205.01 to 2001.97)	170.08 (166.43 to 175.10)
	Mood disorder	*F*	398.24 (359.58 to 436.89)	556.95 (312.56 to 801.34)	489.52 (245.16 to 733.89)	171.08 (166.84 to 175.31)
		M	338.80 (300.71 to 376.89)	413.85 (204.29 to 623.41)	447.02 (230.55 to 663.48)	113.40 (110.04 to 116.77)
	Psychosis	*F*	38.18 (26.30 to 50.06)	125.29 (15.00 to 235.58)	104.90 (0.18 to 209.61)	20.67 (19.26 to 22.08)
		M	41.89 (28.69 to 55.08)	105.95 (2.12 to 209.79)	140.59 (27.71 to 253.47)	26.90 (25.30 to 28.50)
	Bipolar	*F*	26.63 (16.74 to 36.51)	186.67 (57.17 to 316.18)	203.13 (59.38 to 346.88)	64.59 (62.07 to 67.10)
		M	12.56 (5.09 to 20.02)	104.07 (1.76 to 206.38)	114.56 (0.00 to 230.09)	40.04 (38.09 to 42.00)
	Depression	*F*	161.72 (135.36 to 188.07)	312.85 (119.34 to 506.36)	169.12 (17.30 to 320.95)	72.03 (69.21 to 74.86)
		M	159.75 (132.12 to 187.38)	259.84 (90.07 to 429.60)	179.32 (46.07 to 312.57)	53.79 (51.44 to 56.14)
	Antidepressant use	*F*	1469.47 (1359.37 to 1579.56)	7780.32 (6409.21 to 9151.44)	9749.25 (8175.49 to 11323.01)	2787.35 (2766.09 to 2808.61)
		M	1077.03 (989.61 to 1164.45)	7018.84 (5908.32 to 8129.37)	9043.33 (7919.92 to 10166.73)	1719.93 (1705.10 to 1734.76)
	Hypnotic/anxiolytic use	*F*	1177.07 (1093.49 to 1260.65)	6194.26 (5182.31 to 7206.21)	6946.83 (5824.58 to 8069.07)	1234.31 (1221.69 to 1246.93)
		M	994.59 (916.77 to 1072.41)	5887.05 (4977.52 to 6796.59)	7262.96 (6324.08 to 8201.84)	848.07 (838.09 to 858.04)
	Anti-psychotic use	*F*	1141.54 (1076.71 to 1206.37)	6615.04 (5844.21 to 7385.88)	5739.73 (4984.59 to 6494.88)	365.53 (359.80 to 371.25)
		M	1089.97 (1017.97 to 1161.97)	6986.22 (6179.90 to 7792.54)	5363.46 (4674.54 to 6052.38)	344.03 (337.90 to 350.17)

© 2022 Clift AK et al. *JAMA Psychiatry*.

eTable 1. Crude and age-standardized incidence rates (with 95% confidence intervals) per 100,000 person-years for neuropsychiatric sequelae following surviving a hospital admission for severe acute respiratory infections or COVID-19. Follow-up was limited to one year for comparability. Direct standardization was used. F = female sex; M= male

© 2022 Clift AK et al. *JAMA Psychiatry*.

Endpoint of interest	Unadjusted hazard ratio (95% confidence interval)	Age and sex-adjusted hazard ratio (95% confidence interval)	Maximally adjusted (primary analysis; 95% confidence interval)
Anxiety			
SARI	1.88 (1.59 to 2.21)	2.54 (2.12 to 2.96)	1.86 (1.56 to 2.21)
COVID-19	1.92 (1.65 to 2.23)	2.49 (2.12 to 2.86)	2.36 (2.03 to 2.74)
Dementia			
SARI	11.06 (9.43 to 1.96)	2.90 (2.43 to 3.36)	2.55 (2.17 to 3.00)
COVID-19	6.99 (5.88 to 8.32)	2.92 (2.42 to 3.42)	2.63 (2.21 to 3.14)
Psychotic disorder			
SARI	4.49 (2.34 to 8.60)	4.99 (1.74 to 8.24)	3.63 (1.88 to 7.00)
COVID-19	3.97 (2.14 to 7.37)	4.35 (1.66 to 7.03)	3.05 (1.58 to 5.90)
Depression			
SARI	4.40 (2.90 to 6.70)	5.22 (3.02 to 7.42)	3.46 (2.21 to 5.40)
COVID-19	2.15 (1.16 to 3.99)	2.56 (0.99 to 4.13)	1.95 (1.05 to 3.65)
Bipolar affective disorder			
SARI	2.76 (1.57 to 4.84)	3.69 (1.61 to 5.77)	2.26 (1.25 to 4.08)
COVID-19	2.17 (1.24 to 3.82)	2.83 (1.23 to 4.43)	2.26 (1.25 to 4.07)
Antidepressant prescription			
SARI	3.44 (3.05 to 3.88)	3.30 (2.90 to 3.70)	2.55 (2.24 to 2.90)
COVID-19	3.44 (3.10 to 3.81)	3.60 (3.23 to 3.97)	3.24 (2.91 to 3.61)
Hypnotic/anxiolytic prescription			
SARI	6.01 (5.35 to 6.75)	4.45 (3.93 to 4.87)	3.10 (2.74 to 3.51)
COVID-19	5.33 (4.78 to 5.95)	4.56 (4.07 to 5.05)	3.79 (3.38 to 4.25)
Antipsychotic prescription			
SARI	19.58 (17.95 to 21.37)	6.52 (5.92 to 7.12)	4.64 (4.20 to 5.12)
COVID-19	11.91 (10.76 to 13.18)	6.18 (5.55 to 6.81)	4.78 (4.28 to 5.33)

eTable 2. Comparison of unadjusted, age and sex-adjusted, and maximally adjusted hazard ratios (with 95% confidence intervals) from the primary analysis – neuropsychiatric sequelae after discharge from SARI- or COVID-19-related hospitalization, with the remaining population as the reference group.

© 2022 Clift AK et al. *JAMA Psychiatry*.

	Fully adjusted hazard ratio (with 95% confidence interval)	
Endpoint of interest	Primary analysis	Sensitivity analysis
Anxiety		
SARI	1.86 (1.56 to 2.21)	1.99 (1.68 to 2.35)
COVID-19	2.36 (2.03 to 2.74)	2.47 (2.13 to 2.87)
Dementia		
SARI	2.55 (2.17 to 3.00)	2.51 (2.13 to 2.96)
COVID-19	2.63 (2.21 to 3.14)	2.63 (2.20 to 3.14)
Psychotic disorder		
SARI	3.63 (1.88 to 7.00)	3.47 (1.79 to 6.73)
COVID-19	3.05 (1.58 to 5.90)	3.35 (1.77 to 6.34)
Depression		
SARI	3.46 (2.21 to 5.40)	3.50 (2.28 to 5.37)
COVID-19	1.95 (1.05 to 3.65)	2.17 (1.17 to 4.02)
Bipolar affective disorder		
SARI	2.26 (1.25 to 4.08)	2.45 (1.39 to 4.34)
COVID-19	2.26 (1.25 to 4.07)	2.59 (1.47 to 4.56)
Antidepressant prescription		
SARI	2.55 (2.24 to 2.90)	2.53 (2.45 to 2.86)
COVID-19	3.24 (2.91 to 3.61)	3.21 (2.89 to 3.57)
Hypnotic/anxiolytic prescription		
SARI	3.10 (2.74 to 3.51)	3.32 (2.94 to 3.75)
COVID-19	3.79 (3.38 to 4.25)	4.00 (3.59 to 4.47)
Antipsychotic prescription		
SARI	4.64 (4.20 to 5.12)	4.80 (4.36 to 5.28)
COVID-19	4.78 (4.28 to 5.33)	4.95 (4.45 to 5.51)

eTable 3 – Results from sensitivity analyses (maximally adjusted hazard ratios) wherein the reference population group were assigned a random cohort start date to account for varying timeframes in which follow-up was contributed and variation in baseline diagnostic rates during COVID-19 pandemic. Adjustment for calendar month was also performed. Comparison is made with the maximally adjusted hazard ratios from the primary analyses for reference. These analyses were conducted in the contemporary cohort.

© 2022 Clift AK et al. *JAMA Psychiatry*.

	Fully adjusted hazard ratio (with 95% confidence interval)	
Endpoint of interest	Primary analysis	Sensitivity analysis
Anxiety		
SARI	1.86 (1.56 to 2.21)	1.91 (1.62 to 2.26)
COVID-19	2.36 (2.03 to 2.74)	2.38 (1.97 to 2.87)
Dementia		
SARI	2.55 (2.17 to 3.00)	2.55 (1.18 to 2.99)
COVID-19	2.63 (2.21 to 3.14)	2.45 (1.94 to 3.09)
Psychotic disorder		
SARI	3.63 (1.88 to 7.00)	3.38 (1.75 to 6.54)
COVID-19	3.05 (1.58 to 5.90)	2.09 (0.78 to 5.58)
Depression		
SARI	3.46 (2.21 to 5.40)	3.53 (2.24 to 5.56)
COVID-19	1.95 (1.05 to 3.65)	1.83 (0.82 to 4.07)
Bipolar affective disorder		
SARI	2.26 (1.25 to 4.08)	2.29 (1.30 to 4.04)
COVID-19	2.26 (1.25 to 4.07)	2.25 (1.07 to 4.74)
Antidepressant prescription		
SARI	2.55 (2.24 to 2.90)	2.57 (2.28 to 2.90)
COVID-19	3.24 (2.91 to 3.61)	2.70 (2.34 to 3.12)
Hypnotic/anxiolytic prescription		
SARI	3.10 (2.74 to 3.51)	3.17 (2.82 to 3.55)
COVID-19	3.79 (3.38 to 4.25)	3.50 (3.05 to 4.03)
Antipsychotic prescription		
SARI	4.64 (4.20 to 5.12)	4.60 (4.22 to 5.02)
COVID-19	4.78 (4.28 to 5.33)	4.45 (3.91 to 5.08)

eTable 4 – Maximally adjusted hazard ratios obtained from sensitivity analyses excluding COVID-19-related hospitalization survivors that did not have COVID-19 as the primary reason for admission to hospital (e.g. those that had a positive SARS-CoV-2 test within the preceding 14 days, but did not explicitly have COVID-19 as the primary reason for hospital admission). These analyses were conducted in the contemporary cohort.

© 2022 Clift AK et al. *JAMA Psychiatry*.
